# Synergizing liver systemic treatments with interventional oncology: friend or foe?

**DOI:** 10.1259/bjr.20220548

**Published:** 2022-09-14

**Authors:** Raphaël Jost, Nael Al-Shatti, Mario Ghosn, Baptiste Bonnet, Stephane Champiat, Frederic Deschamps, Maximiliano Gelli, Valérie Boige, Francois-Xavier Danlos, Sandrine Susini, Antoine Hollebecque, Samy Ammari, Aurelien Marabelle, Thierry de Baere, Lambros Tselikas

**Affiliations:** 1 Département d’Anésthésie, Chirurgie et Imagerie Interventionnelle, Gustave Roussy, Villejuif, France; 2 Centre d’Investigation Clinique BIOTHERIS, INSERM CIC1428, Villejuif, France; 3 Laboratoire de Recherche Translationnelle en Immunothérapie (LRTI), INSERM U1015, Villejuif, France; 4 Département d’Innovation Thérapeutique et d’Essais Précoces (DITEP), Gustave Roussy, Villejuif, France; 5 Department of medical oncology, Gustave Roussy, Villejuif, France; 6 Faculté de Médecine, Université Paris Saclay, Le Kremlin-Bicetre, France; 7 Département de Radiologie, Gustave Roussy, Villejuif, France

## Abstract

Interventional radiology techniques provide excellent local tumor control for small tumors in various organs, but several limitations can hamper the oncological outcomes such as the tumor size or the number of lesions. Technical improvements, optimal patient selection and combination with systemic therapies, including immune checkpoint inhibitors, have been successfully developed to overcome these barriers.

In this setting, chemotherapy and targeted therapies aim to diminish the tumor burden in addition to local treatments, while immunotherapies may have a synergistic effect in terms of mechanism of action on the tumor cell as well as the immune environment, with multiple treatment combinations being available. Finally, interventional Rrdiology treatments often increase tumor antigen exposure to the immune system, and thus stimulate a specific antitumor immune response that can act beyond the treated site. Notwithstanding their many benefits, combination treatment may also result in complications, the most feared may be auto-immune-related adverse events.

In early studies, several combined therapies have shown promising levels of safety and efficacy, particularly in hepatocellular carcinoma.

This review provides a comprehensive and up-to-date overview of results of combined therapies for primary and secondary liver malignancies. Recent advances and future perspectives will be discussed.

## Introduction

Interventional radiology (IR), through image-guided locoregional therapies (LRTs) is now the fourth pillar of cancer care with medical oncology, surgery and radiation therapy, especially for liver cancer.^1^ IR minimal invasive approaches preserve the liver function without compromising the treatment’s efficacy, while image-guidance ensures safe and precise tumor targeting for both percutaneous and intravascular approaches.^2^ In liver cancer, LRTs play a major role for both local and advanced stages, although their efficacy can be compromised by tumors’ size and number.^3^

A growing body of evidence suggests that LRTs might play a critical synergistic role in combination to immunotherapy, targeted therapy (TT) and others systemic treatments (SYST). Indeed, LRTs can trigger both local and systemic immune-responses that are mediated by checkpoint proteins and local release of tumor-associated-antigens (TAA).^4^

Recently, the combination of LRTs and SYST has offered new perspectives for the management of liver cancer.^5^ Indeed, combined therapies of LRTs and SYST might provide a synergizing antitumoral effect that encompasses the activity of each of these therapies when used alone. Thereby, these combinations might generate a systemic and a local meaningful antitumor response or even prevent tumor recurrences. Additionally, combination treatments may help downstaging tumors before surgical resection or improve treatments safety. However, combined therapies of LRTs and SYST have not been well established yet because some randomized control trials (RCTs) resulted in negative results, while others are still ongoing.

The purpose of this review is to provide an updated insight into the potential role of IR treatments in combination with systemic therapies for primary and secondary liver malignancies. Rationale of these combinations and results of clinical trials will be presented and discussed along with future directions.

## Primary liver cancer

### Hepatocellular carcinoma

Primary liver cancer is the sixth most commonly diagnosed cancer and the third cause of death by cancer worldwide, with an increasing incidence.^6,7^ The most frequent histological types are hepatocellular carcinoma (HCC) (75–85% of cases) and intrahepatic cholangiocarcinoma (iCCA) (10–15%).^8,9^ The complex management of HCC requires a multidisciplinary approach.^7,10^ Although early surgical resection and liver transplantation may be the best treatments to ensure full recovery, they can only be proposed to a minority of selected patients and are limited by the number of available liver transplants.^11^ Indeed, many patients with HCC are diagnosed at an intermediate or advanced stage, as early forms of disease commonly remain clinically silent and are only diagnosed in the setting of systematic surveillance of cirrhosis. For these patients, representing approximately 80% of all HCCs, LRTs may play a leading role.^7,12–14^ A significant improvement in prognosis has been achieved over time, and mainly during the last decades, with median overall survival (OS) times of approximately 6 *vs* 3 years for early stages, 26–30 months *vs* 16 months for intermediate stages and 20 *vs* 6 months for advanced stages.^7^ In particular, a significant increase in OS has been obtained with SYST.^15^ For instance, TT have demonstrated a significant OS improvement in RCT, resulting in their approval by both the Food and drugs administration (FDA) and European medicines agency.^16^

Given the importance of LRTs as well as immune and targeted SYST in HCC management,^10^ the option of combining both became of particular interest,^17^ and is under evaluation as first- or second-line therapies, but also in the neoadjuvant and adjuvant setting.

### LRTs and immune status

HCC alters the immune balance of patients^18^ and can cause local humoral and cellular immune responses in the liver tumor microenvironment (TME).^19^ While spontaneous immune responses have been described and may be related to the presence of TAA,^20^ HCC has several mechanisms to evade the host’s immune response. Indeed, HCC progression triggers many immune regulatory changes that promote tumor tolerance and thus its subsequent progression.^9^ For instance, near TME, HCC produces acidity which neutralizes the activity of antitumor immune effectors and activates the regulatory immune cells (Tregs). In addition, TME hypoxia caused by HCC annihilates the T-cells function and their immunologic roles.^19^ More precisely, T-cells in the TME are in a hyporesponsive state as they become exhausted after overexpressing inhibitory receptors such as cytolytic T-lymphocyte-associated protein 4 (CTLA-4) or programmed death-1 (PD-1).^21^ T-regs cells CD4+CD25+ increase in the HCC TME^22^ and in the peripherical blood.^23^ All of these immunological changes lead to the reduction of production of proinflammatory cytokines as IFN-γ or TNF-α while increasing the secretion of immunosuppressive cytokines as IL-4 or IL-10 in the TME.^24^ Myeloid-derived suppressor cells (MDSCs) also play a major role in tumor immunotolerance as they can negatively influence cytotoxic T-cells population and expand the population of T-regs and consequently boost of the host’s immunosuppressive activity.^25^

The goal of immunotherapy is to overcome the cancer immune-tolerance and create an efficient T-cell-mediated adaptative antitumor immune response. Use of immunotherapy is, however, still limited to advanced HCC stages with a low rate of complete responses.^26^ The combination with LRTs both in early and advanced stages could help bypassing the TME obstacle by priming locally an immune response.

Indeed, the immune system may react to LRTs and subsequent cell death.^27^ LRTs lead to cell death by hypoxia with Transarterial Chemoembolization (TACE) or necrosis with percutaneous ablation. The intracellular content of cells, various TAA and tumor neoantigens are subsequently released and prime a non-infectious inflammatory immune response. This ability to unmask TAA facilitates the recruitment, proliferation and activation of tumor-specific T-cells and antigen-presenting cells (APCs) such as dendritic cells (DCs) into the TME.^28,29^

Although LRTs induce a systemic-specific immune response, as described in many series,^30,31^ this has not yet translated into clinically meaningful effects.^32^ Indeed, LRTs can also cause a pro-tumorigenic effect. For instance, periablation zone caused by radiofrequency ablation (RFA) or microwave ablation (MWA) increase the production of VEGF, IL-6, hepatocyte growth factor or c-met in liver regeneration and promote the growth of local or distant metastases.^33–35^ Blocking c-met or vascular endothelial growth factor (VEGF) might suppress these effects and promote a synergizing benefit of LRTs combined with SYST.^36^ Therefore, local and systemic inflammation could be decreased or regulated by adding TT or immunotherapy.^37^

### Very early stage (0) and early stage (A)

Three main strategies have been included into the guidelines, with a curative objective: resection with hepatectomy, local ablation, and liver transplantation.^10,13^ Other therapies such as superselective TACE, or selective internal radiation therapy (SIRT) can also be proposed as down-taging strategies in order to permit liver transplantation.^10,13,38^

The most used local ablation technique is RFA. It represents the first-line curative treatment for single tumors <2 cm, *i.e*. very early stage (0), or an alternative to surgery for a single 2–5 cm lesion or a multinodular disease (2–3 lesions, each <3 cm). MWA is an option, already promoted by USA guidelines^13^ but not yet by European guidelines^10^ as data from Phase III RCT are lacking. Other ablatives techniques such as irreversible electroporation (IRE) or cryoablation (CA), are currently under investigation.^10,13^

According to the STORMphase III trial,^39^ the combination of surgical resection or RFA with sorafenib as adjuvant therapy did not improve recurrence-free-survival (RFS) (33.3 months with sorafenib and 33.7 months with placebo, *p* = 0.26) and resulted in more severe AEs noted for the sorafenib group (52% vs 10%). Consequently, no adjuvant therapy is currently recommended following ablation.

The HEAT study^40^ investigated RFA plus intravenous lyso-thermosensitive liposomal doxorubicin (LTLD). No differences were observed in terms of PFS (median PFS : 13.9 months for both) and OS (median OS : 53.7 and 53.4 months for combined therapy and RFA alone respectively, *p* = 0.67).

In this field, the results of the ongoing OPTIMA study (NCT02112656) may be of significant interest. It explores the LTLD combined with RFA (lasting >45 min) for a single lesion of 3–7 cm in comparison to RFA alone.

Clinical trials have studied the combination of cytokine-induced killer (CIK) with RFA^41^ and RFA or percutaneous ethanol injection (PEI) or surgery^42^ and yielded interesting oncological results.

Bian et al showed the combination of 131I-metuximab with RFA led to a longer time to recurrence than RFA alone (median time to tumor recurrence: 17 *vs* 10 months, *p* = 0.03).^43^

Adjuvant immune checkpoint inhibitors (ICI) and vascular targeted agents might be used after a local ablation as in the EMERALD-2 (NCT03847428) trial which is comparing three combinations of durvalumab and/or bevacizumab and/or placebo after resection or local ablation.

Encouraging preliminary results of the NIVOLVE trial (NCT000026648) showed that the combination of RFA or surgical resection with nivolumab as an adjuvant therapy reached 76.7% recurrence-free survival rate at 1 year and a median of 26 months of recurrence-free survival.

Other Phase II or III studies using ICI and/or targeted therapies as neoadjuvant and/or adjuvant treatment after a local ablation or a combination of local ablation with intravascular treatment are underway Tables 1; 2 Indeed, many immunological effects have been suggested in patients undergoing LRTs, not seen in surgical resection patients.^9^

**Table 1.** Trials on LRTs and SYST combinations for stage (A) HCC

Trial/First author	NCT	Year	Phase	Clinical scenario	Treatment arms	N	Primary end point	Outcome
Lee	00699816	2018	III	Undergone curative treatment (surgery, RFA, or PEI)	CIK + RFA or PEI or Surgery RFA or PEI or Surgery	162	RFS	5 year RFS combination arm: 44.8% 5 year RFS control arm: 33.1% h: 0.67; 95% CI: 0.48–0.94; **P: 0.01** Median OS: not reached HR: 0.33; 95% CI: 0.15–0.76; **P: 0.006**
HEAT/Tak	00617981	2018	III	HCC unresectable and < 4 HCC and at least one HCC: 3.0–7.0 cm	LTLD + RFA RFA	354	PFS	Median PFS combination arm: 13.9 *mo* Median PFS control arm: 13.9 *mo* HR: 0.96; 95% CI: 0.79–1.18; **P: 0.71** Median OS combination arm: 53.7 *mo* Median OS control arm: 53.4 *mo* HR: 0.95; 95% CI: 0.76–1.20; **P: 0.67**
STORM/Bruix	00692770	2015	III	HCC with a complete radiological response after surgical resection or local ablation	Surgery or local ablation + sorafenib Surgery or local ablation + placebo	1602	RFS	Median follow-up for RFS combination arm: 8.5 *mo* Median follow-up for RFS placebo: 8.4 *mo* Median RFS combination arm: 33.3 *mo* Median RFS placebo arm: 33.7 *mo* HR: 0.940; 95% CI 0.780–1.134; **one-sided P: 0.26**
Lee	00699816	2015	III	Undergone curative treatment (surgery, RFA, or PEI)	CIK + RFA or PEI or Surgery RFA or PEI or Surgery	230	RFS	Median time of RFS combination arm: 44.0 *mo* Median time of RFS control arm: 30.0 *mo* HR: 0.63; 95% CI: 0.43–0.94; **P: 0.010** Median OS: not reached HR: 0.21; 95% CI: 0.06–0.75; **P: 0.008**
Bian	Na	2014	II	BCLC 0, A or B	131I-metuximab + RFA RFA	127	OR	Median time to OR combination arm: 17 *mo* Median time to OR control arm: 10 *mo* HR: 0.60, 95% CI: 0.38–0.96, **P: 0.03**
Cui	30901702	2014	I/II	HCC: 2.0–8.0 cm and < 5 HCC	CIK + RFA RFA	62	PFS	Median PFS combination arm: not reached Median PFS control arm: 12.0 *mo* HR: 0.136; 95% CI: 0.049–0.379; ***p* < 0.0001**

BCLC: Barcelona-Clinic-Liver Cancer; CIK: Cytokine-induced killer; HCC: Hepatocellular carcinoma; LTLD: Lyso-thermosensitive liposomal doxorubicin; N: Number of patients; Na: Not applicable; NCT: National Clinical Trial; OR: Overall recurrence; PEI: Percutaneous ethanol injection; PFS: Progression-free survival; RFA: Radiofrequency ablation; RFS: Recurrence free survival.

**Table 2.** Ongoing trials on LRTs and SYST combinations for stage (A) HCC

Trial/First author	NCT	Phase	Clinical scenario	Treatment arms	N	Primary end point	Outcome
CheckMate9DX	03383458	III	HCC with a complete radiological response after surgical resection or local ablation with high risk of recurrence	Surgery or local ablation + nivolumab Surgery or local ablation + placebo	530	RFS	In progress
EMERALD-2	03847428	III	HCC with a complete radiological response after surgical resection or local ablation with high risk of recurrence	Surgery or local ablation + durvalumab+bevacizumab Surgery or local ablation + durvalumab+placebo Surgery or local ablation + placebo+placebo	888	RFS	In progress
IMMULAB	03753659	II	HCC ⩽ 7.0 cm and ⩽ 5 HCC	Pembrolizumab + RFA and/or MWA and/or Brachytherapy Pembrolizumab + TACE+ RFA and/or MWA and/or Brachytherapy	30	ORR	In progress
AB-LATE02	04727307	II	HCC ⩽ 3.0 cm and ⩽ 3 HCC	Atezolizumab + Bevacizumab+ RFA RFA	202	RFS	In progress
NIVOLEP	03630640	II	Solitary HCC: 2.0–5.0 cm or HCC ⩽ 3 cm and ⩽ 3 HCC	Nivolumab + IRE	43	RFS	In progress
KEYNOTE-937	03867084	II	Undergone curative treatment (surgery or RFA)	RFA or surgery + pembrolizumab RFA or surgery + placebo	950	RFS OS	In progress
IMbrave 050	04102098	III	Undergone curative treatment (surgery or RFA)	RFA or surgery + atezolizumab+bevacizumab Active surveillance	662	RFS	In progress
NIVOLVE	000026648	II	Undergone curative treatment (surgery, RFA) Child–Pugh class A cirrhosis	RFA or Surgery + nivolumab (adjuvant)	55	RFSR	Preliminary results: 1 year RFSR: 76.7% Median RFS: 26.0 *mo* (95% CI, 23.9–28.1), with no difference between SR and RFA
OPTIMA	02112656	III	HCC: 3.0–7.0 cm	RFA + LTLD RFA + Placebo	556	OS	In progress

HCC: Hepatocellular carcinoma; LTLD: Lyso-thermosensitive liposomal doxorubicin; MWA, Microwave ablation; N: Number of patients; Na: Not applicable; NCT: National Clinical Trial; OR: Overall recurrence; PEI: Percutaneous ethanol injection; PFS: Progression-free survival; RFA: Radiofrequency ablation; RFS: Recurrence free survival.

For several reasons,^33–35^ the risk of intrahepatic or distant recurrence persists even after RFA. Given the potential role of atezolizumab plus bevacizumab for advanced stage disease,^44^ AB-LATE02 is exploring the neoadjuvant/adjuvant strategy for RFA treated patients in the hope of reducing the pro-oncogenic effects of RFA and so the risk of recurrence (RFS). The AB-LATE02 trial (NCT04727307, Table 2.) is investigating in the first arm the combination of atezolizumab (as a neoadjuvant treatment) and RFA followed by atezolizumab plus bevacizumab (as an adjuvant treatment). In the other arm, patients are treated by RFA alone.

### Intermediate stage (B)

Conventional transarterial chemoembolization (cTACE) with Lipiodol^®^, drug-eluting beads transarterial chemoembolization (DEB-TACE), transarterial radioembolization (TARE) and hepatic arterial infusion of chemotherapy (HAIC) represent the LRTs arsenal for intermediate stage. BCLC B stage is heterogeneous explaining the large spectrum of OS in different studies. Following the results of several RCT,^45,46^ TACE has become the main therapeutic approach for this stage.^10,13^

TARE and cTACE may share consistent efficacy, as suggested by Phase II randomized trial,^47^ with time-to-progression (TTP) of more than 26 months for SIRT *vs* 6.8 months for cTACE but no statistically significant difference for OS (18.6 months *vs* 17.7 months, respectively). More recently, the TRACE study^48^ showed an improvement of TTP and OS in favor of TARE *vs* DEB-TACE, with a similar safety profile. Consequently, TARE may be a therapeutical option, as mentioned in the guidelines, but with a lower level of evidence.^10,13^

Various Phase II/III randomized trials^49–53^ have investigated the combination of TACE with time-to-progression (TKI) (Sorfenib, Orantinib or Brivanib) and have not shown any substantial clinical benefits for the combination compared to TACE alone.

In addition, the embolization in TACE procedure triggers acute tissue hypoxia in part of the targeted liver leading to a proangiogenic growth factor activation such as transient increase of VEGF which could be targeted by TKI.^54^

Despite this assumption, Pinter et al did not demonstrate any significant improvement in ORR and OS when combining cTACE with bevacizumab, while reporting a higher risk of severe sepsis and vascular complications.^55^ According to Pinter et al, bevacizumab might not be recommended as an adjuvant treatment for cTACE.

SPACE trial^51^ investigated the combination of DEB-TACE plus sorafenib. The combination was technically feasible, but median TTP was not statistically significantly different between the two arms (169 *vs* 166 days, respectively, *p* = 0.072). Of note, modified Response Evaluation Criteria in Solid Tumors (mRECIST) was used to define radiological progression.

Recently, the TACTICS trial showed a significantly improved PFS (25.2 *vs* 13.5 months, *p* = 0.006)^56^ for the combination of TACE plus sorafenib. Times to vascular invasion, extrahepatic spread and stage progression were longer for the combined therapy. The particularity of the TACTICS study was how authors defined disease progression. Indeed, untreatable (UnTACEable) progression was defined as the patient inability to be treated again by TACE. Intrahepatic tumor progression based on the Response Evaluation Criteria in Cancer of the Liver (RECICL)^57^ could be a reason to be “UnTACEeable”. For the authors, RECIST 1.1 or mRECIST criteria are inappropriate to evaluate tumor progression in patients treated with TACE.

Of note, patients included in the TACTICS trial were treated with sorafenib for a longer time than in the SPACE trial probably due to the use of the RECICL criteria which allow patients to have a longer duration before assessing tumor status progression. Therefore, the combination of TACE plus sorafenib could prevent the progression of intermediate to advanced stage HCC.

In another study, Li et al*^58^* suggested that the combination of DEB-TACE plus Apatinib might enhance significantly OS and PFS with similar adverse effects compared to TACE alone in ≥10 cm HCC lesions.

Other studies have suggested Lenvatinib as a neoadjuvant therapy followed by TACE for initial patients deemed TACE-ineligible.^45,59^

Overall, despite the TACTICS results, data supporting sorafenib or other TKI treatments in combination with TACE are still not solid enough for this association to be considered reliable. These results have led to the combination of LRTs with ICI such as PD-1 inhibitors in the hope that such association could improve outcomes in stage (B) patients. Preliminary results of the Phase I/II PETAL trial (NCT03397654) showed high tolerability of pembrolizumab after TACE without cumulative adverse effects (AE). Other trials testing ICI with or without TKI as a neoadjuvant or adjuvant treatment in combination with LRTs are currently ongoing Tables 3; 4.

**Table 3.** Trials on LRTs and SYST combinations for stage (B) HCC

Trial/First author	NCT	Year	Phase	Clinical scenario	Treatment arms	N	Primary end point	Outcome
Li	Na	2022	Retrospective	HCC ⩾ 10.0 cm	DEB-TACE (pirarubicin) + apatinib DEB-TACE (pirarubicin)	73	PFS OS	Median PFS combination arm:19.0 *mo* Median PFS control arm: 10.9 *mo* HR: 0.420; 95% CI: 0.232–0.761; **P: 0.004** Median OS combination arm: 25.1 *mo* Median OS control arm: 13.7 *mo* HR: 0.477; 95% CI: 0.253–0.898, **P: 0.022**
TACTICS/Kudo	01217034	2020	II	Unresectable HCC not suitable for curative treatment	TACE + sorafenib TACE	176	PFS OS	Median PFS combination arm: 25.2 *mo* Median PFS control arm: 13.5 *mo* HR: 0.59; 95% CI, 0.41–0.87; **P: 0.006** Median TTUP combination arm: 26.7 *mo* Median TTUP combination arm: 20.6 *mo* HR: 0.57; 95% CI: 0.36–0.92; **P: 0.02** Median TTP combination arm: 26.7 *mo* Median TTP ccombination arm: 16.4 *mo* HR:0.54; 95% CI: 0.35–0.83; **P: 0.005**
ORIENTAL/Kudo	01465464	2018	III	Unresectable HCC not suitable for curative treatment	TACE + orantinib TACE + placebo	889	OS	Median OS combination arm: 31.1 *mo* Median OS control arm: 32.3 *mo* HR: 1.090; 95% CI: 0.878–1.352; **P: 0.435**
TACE-2/Meyer	93375053	2017	III	Unresectable HCC not suitable for curative treatment	DEB-TACE (doxorubicin) + sorafenib DEB-TACE (doxorubicin) + placebo	313	PFS	Median PFS combination arm: 238 *days* Median PFS control arm: 235 *days* HR: 0.99; 95% CI: 0.77–1.27; **P: 0·94** Median OS combination arm: 631 *days* Median OS control arm: 598 *days* HR: 0.91; 95% CI: 0.67–1.24; **P: 0.57**
SPACE/Lencioni	00855218	2016	II	Multinodular HCC	DEB-TACE (doxorubicin) + sorafenib DEB-TACE (doxorubicin) + placebo	307	TTP	Median TTP combination arm: 169 *days* Median TTP placebo arm: 166 *days* HR: 0.797; 95% CI: 0.588–1.080; **one-sided P: 0.072**
Pinter	Na	2015	II	BCLC C	cTACE (doxorubicin) + bevacizumab cTACE (doxorubicin) + placebo	32	Progression at 12 *mo*	Median TTP combination arm: 7.2 mo Median TTP control arm: 11.7 mo HR: 0.9; 95% CI: 0.3–2.4; **P: 0.754** Median OS combination arm: 5.3 *mo* Median OS combination arm: 13.7 *mo* HR: 1.7; 95% CI: 0.8–3.6; **P: 0.195**
BRISK/Kudo	00908752	2014	III	Unresectable HCC not suitable for curative treatment	TACE + brivanib TACE + placebo	502	OS	Median OS combination arm: 26.4 *mo* Median OS control arm: 26.1 *mo* HR: 0.90; 95% C: 0.66–1.23; **log-rank P: 0.5280**
Kudo	Na	2011	III	BCLC B responsed with TACE	TACE + sorafenib TACE + placebo	458	TTP	Median TTP combination arm: 5.4 *mo* Median TTP placebo arm: 3.7 *mo* HR: 0.87; 95% CI: 0.70–1.09; **P: 0.252** Median OS combination arm: 29.7 *mo* Median OS placebo arm: Not reached HR: 1.06; 95% CI: 0.69–1.64; **P: 0.79**

BCLC: Barcelona-Clinic-Liver Cancer; cTACE: Conventional transarterial chemoembolization; DEB-TACE: Drug-eluting bead-transarterial chemoembolization; HCC: Hepatocellular carcinoma; N: Number of patients; Na: Not applicable; NCT: National Clinical Trial; OS: Overall survival; PFS: Progression-free survival; RFS: Recurrence free survival; TACE: Transarterial chemoembolization; TTP: Time-to-progression; TTUP: Time to untreatable progression.

**Table 4.** Ongoing trials on LRTs and SYST combinations for stage (B)

Trial/First author	NCT	Phase	Clinical scenario	Treatment arms	N	Primary end point	Outcome
Na	03869034	II	Potentially resectable Locally advanced HCC	HAIC-FOLFOX + sintilimab HAIC-FOLFOX	40	PFS	In progress
EMERALD-1	03778957	III	Unresectable HCC not suitable for curative treatment	TACE + durvalumab+placebo TACE + durvalumab+bevacizumab TACE + placebo+placebo	724	PFS	In progress
LEAP-012	04246177	III	Unresectable HCC not suitable for curative treatment	TACE + lenvatinib+pembrolizumab TCA + placebo+placebo	950	PFS	In progress
ML-42612	04712643	III	Unresectable HCC not suitable for curative treatment	TACE + atezolizumab+bevacizumab TACE	342	PFS OS	In progress
TACE-3	04268888	II/III	Unresectable HCC not suitable for curative treatment	TACE/TAE + nivolumab TACE/TAE	522	OS TTTP	In progress
Na	04229355	III	Unresectable HCC not suitable for curative treatment	DEB-TACE + sorafenib DEB-TACE + lenvatinib DEB-TACE + PD-1 inhibitor	90	PFS	In progress
CHECKMATE-74W	04340193	III	BCLC B	TACE + nivolumab+ipilimumab TACE + nivolumab TACE	26	Number of AEs Number of deaths Laboratory abnormalities	In progress
IMMUTACE	03572582	II	Unresectable HCC	TACE + nivolumab	49	ORR	In progress
PETAL	03397654	II	Unresectable HCC	TACE + prembrolizumab	26	Incidence of AEs	In progress
Na	03143270	I	Unresectable HCC	DEB-TACE + Nivolumab	14	Incidence of AEs	In progress
Na	04220944	I	BCLC B or C HCC ⩾ 5.0 cm	MWA + TACE+Sintilimab	45	PFS	In progress
Na	03864211	I/II	BCLC B	RFA or MWA + toripalimab Toripalimab	147	PFS	In progress
Na	03638141	II	BCLC B	DEB-TACE + durvalumab+tremelimumab	30	PFS	In progress
Na	04174781	III	Patients with BCLC A but beyond Milan Criteria and BCLC stage B not eligible for primary resection or local ablation	DEB-TACE + sintilimab	61	PFS	In progress
Na	04472767	II	Unresectable HCC	cabozantinib + ipilimumab+nivolumab + TACE	35	PFS	In progress
ROWAN	05063565	II	Unresectable HCC	TARE(Y90) + tremelimumab + durvalumab TARE(Y90)	150	ORR DoR	In progress
ASCO	04541173	II	BCLC B HCC and must be outside of downstaging criteria	TARE(Y90) + bevacizumab + atezolizumab TARE(Y90)	128	PFS	In progress
SOLID	04124991	I/II	Unresectable HCC	TARE(Y90) + durvalumab	24	TTP	In progress
NASIR-HCC	03380130	II	Unresectable HCC	TARE(Y90) + nivolumab	42	Safety	Median follow up 7.8 *mo* (2.5–16.3) ORR = 40% Serious AEs: 12%
Na	04150744	II	BCLC B or C	RFA + carrizumab RFA + placebo	120	PFS	In progress
EMERALD-3	05301842	III	Unresectable hepatocellular carcinoma not suitable for curative treatment	TACE + tremelimuma+durvalumab + lenvatinib TACE + tremelimuma + durvalumab TACE	525	PFS	In progress

AEs: Adverse effects chemoembolization; BCLC: Barcelona-Clinic-Liver Cancer; DoR: Duration of reponse; HCC: Hepatocellular carcinoma; MWA: Micro-wave ablation; N: Number of patients; Na: Not applicable; NCT: National Clinical Trial; ORR: Objective response rate; OS: Overall survival; PFS: Progression free survival; TACE: Transarterial chemoembolization; TAE: Transarterial embolization; TARE: Transarterial radioembolization; TTP: Time-to-progression.

It is worth mentioning that intermediate stage HCC includes a highly heterogeneous patient population with a wide range of tumor burden and/or different liver functions. In this regard, subclassifications of intermediate stage has been proposed by Bolondi et al^46^ and Kudo et al with Kinki criteria.^60^ In light of recent trials, stage B2 and B3 of Bolondi’s subclassification might be ideal candidates for combination therapy using TACE.

Given this heterogeneity, a personalized approach remains a key factor to improve patients’ outcomes

#### Advanced stage (C)

The management of advanced stage mostly consists of SYST, either alone or in combinations.^10,13^

SARAH and SIRveNIB are Phase III trials which compared SIRT with Sorafenib in BCLC stage B or C patients. While demonstrating no meaningful improvements in OS, the SIRveNIB trial showed a more favorable toxicity profile in the SIRT treatment arm, with an AE ≥3 rate of 27.7%, compared to 50.6% in the Sorafenib group.^61,62^

This was further explored in the Phase III SORAMIC trial, where a non-statistically significant improvement in OS was observed when combining SIRT with Sorafenib (12.1 *vs* 11.4 months for sorafenib alone, *p* = 0.9529), with an AE ≥3 rate of 64.8%, compared to 53.8% in the sorafenib-only group.^63^ Additionally, a recent meta-analysis confirmed the non-inferiority of SIRT to Sorafenib for stage (C) in terms of OS while offering a better safety profile.^64^ Therefore, while waiting for the results of the STOP-HCC trial (NCT01556490), the combination of SIRT with Sorafenib is not currently recommended.

Interestingly, a subgroup analysis of the SORAMIC trial identified a survival benefit in patients under 65 years, as well as non-cirrhotic patients, or patients suffering from non-alcoholic cirrhosis.^63^ This in-turn prompted further investigation regarding radiation dose optimization when combining SIRT with TKI, or ICI, in selected patients. Garin et al has recently compared personalized dosimetry, ≥205 Gy targeted at the index lesion, with the standard approach of 120 ± 20 Gy targeted at the perfused lobe, in locally advanced HCC. The comparison showed an ORR of 71% in the personalized dosimetry group, compared to only 36% in the standard irradiation group, with an OS and PFS of 26.6 and 6 months respectively in the experimental arm, *vs* 10.7 (*p* = 0.0096) and 3.4 (*p* = 0.26) months in the latter group.^65^

As for TACE, a recent Phase III trial (STAH) demonstrated that the combination of TACE and Sorafenib did in fact prolong TTP, PFS, and improve tumor response rates, despite no statistically significant improvements in OS (12.8 months *vs* 10.8 months for sorafenib alone, *p* = 0.290).^66^

These results were confirmed in an additional retrospective study, demonstrating a significantly improved TTP of 7 months in the RFA, TACE, and Sorafenib treatment group in stage (C) patients following initial hepatectomy, and an OS of 14 months, compared to a TTP and OS of 4 and 9 months respectively in the Sorafenib only group.^67^ Furthermore, according to LAUNCH trial,^68^ the combination of TACE and Lenvatinib improves OS and PFS. Indeed, this combination showed a significant OS improvement (17.8 *vs* 11.5 months, *p* < 0.001). Hence, this combination might be a potential treatment for advanced stage disease.

In a recent Phase III trial named HAIC-FO,^69^ hepatic arterial infusion chemotherapy (HAIC) demonstrated better OS compared to sorafenib in advanced stage (13.9 *vs* 8.2 months, *p* < 0.001). On one hand, HAIC has also been tested by a Phase III trial, SILIUS,^70^ which did not demonstrate an improvement by combining HAIC (cisplatin and fluorouracil) with sorafenib in terms of OS (11.8 *vs* 11.5 months for sorafenib alone, *p* = 0.955) although TTP and response rate were superior in the combination arm. On the other hand, the combination of HAIC and sorafenib demonstrated an OS improvement compared with sorafenib alone in a Phase II trial (10.6 *vs* 8.7 months, *p* = 0.031)^71^ and a Phase III trial (13.37 *vs* 7.13 months (*p* < 0.001)^72^ . Of note, Ikeda et al only included patients with portal vein invasion.^71^

As for anti-VEGF therapy, a recent meta-analysis revealed that TACE in combination with Apatinib is both effective and safe in advanced stage, with an OS of 10.0 months in the combined group, compared to 6.2 months in the TACE alone group (*p* = 0.01).^73^ Similarly, combined DEB-TACE and Apatinib have been also shown to improve OS and PFS.^74^

As for translational results, it has been recently shown that a CD8^+^ T cell response against TAA is associated with improved RFS in HCC patients following ablation, meaning that ablation therapy can potentially transform non-infiltrated “cold” tumors into more immunogenic “warm” tumors. This indicates a potential synergy between the immunological impact of local ablation and immunotherapy.^75^ This was explored by Duffy et al, demonstrating a potential clinical benefit of combining Tremelimumab, an anti-CTLA4 antibody, with subtotal ablation or TACE in advanced stage, pre-treated patients with HCC. Tumor biopsies have further demonstrated a strong correlation between antitumor response and CD8+ tumor infiltration.^76^

A current trial exploring the safety and efficacy of TARE combined with Nivolumab in advanced stage has demonstrated an excellent safety profile, with 9 out of 11 patients achieving disease control.^77^ This was shortly followed up by a Phase II counterpart, with encouraging preliminary results, including an ORR of 30% and a median OS and PFS of 15.1 and 4.6 months, respectively.^78^ Consequently, several ongoing trials are assessing combinations of LRTs and ICI Tables 5; 6

**Table 5.** Trials on LRTs and SYST combinations for stage (C) HCC

Trial/First author	NCT	Year	Phase	Clinical scenario	Treatment arms	N	EHS (%)	VI (%)	Primary end point	Outcome
LAUNCH/Peng	03905967	2022	III	BCLC C	TACE + Lenvatinib Lenvatinib	338	Na	Na	OS	Median OS combination arm: 17.8 *mo* Median OS control arm: 11.5 *mo* HR: 0.45, 95% CI 0.33–0.61, ***p* < 0.001** Median PFS combination arm: 10.6 *mo* Median PFS control arm: 6.4 *mo* HR: 0.43; 95% CI: 0.34–0.55; ***p* < 0.001**
Ju	Na	2022	Retrospective	BCLC C unable to receive palliative surgery or radiation therapy	DEB-TACE + apatinib	110	Na	110 (100)	OS PFS	3 and 6 months ORR: 77.2 and 59.1% Median PFS: 6.3 *mo* (95% CI: 5.0–7.7) 1, and 2 year PFS: 19.8, 3.3% Median OS: 16.9 *mo* (95% CI: 10.2–23.7) 1, 2 and 3 year OS: 66.5, 34.7 and 14.2%
FOHAIC-1/Lyu	03164382	2022	III	BCLC C	HAIC (oxaliplatin + fluorouracil) Sorafenib	262	90 (34)	185 (70)	OS	Median OS HAIC arm: 13.9 mo Median OS sorafenib: 8.2 mo HR: 0.408; 95% CI: 0.301–0.552; ***p* < 0.001**
Liu	Na	2021	Retrospective	BCLC C	TACE + apatinib TACE	146	62 (42)	88 (60)	OS	Median TTP combination arm: 5.5 *mo* Median TTP control arm: 3.7 *mo* ***p* < 0.02** Median OS combination arm: 10.0 *mo* Median OS control arm: 6.2 *mo* ***p* < 0.01**
Fenton	02837029	2021	I	BCLC C	SIRT + nivolumab	27	Na	Na	Maximum Tolerated Dose	Tolerable and a maximum tolerated dose of nivolumab established Clinical benefit rate of 82%: 9/11 patients achieving stable disease
Tai/CA-209–678	03033446	2021	II	BCLC C ineligible for curative surgery	TARE(Y90) + nivolumab	36	13 (36)	16 (44)	ORR	ORR: 30.6% (95% CI: 16.4–48.1) Median PFS: 4.6 *mo* (95% CI: 2.3–8.4) Median OS: 15.1 *mo* (95% CI 7.8-not reached)
NEMESIS/Venerito	Na	2020	meta-analysis	BCLC C	SIRT ± sorafenib Sorafenib	933	Na	Na	OS Severe AEs	Median OS combination arm: 10.2 *mo* Median OS control arm: 9.2 *mo* HR: 0.91; 95% CI: 0.78–1.05; **P: Na** Severe AEs combination arm: 149/515 (28.9%) Severe AEs control arm: 249/575 (43.3%) Risk Difference: −0.14; 95% CI: −0.20–−0.09; ***p* < 0.01**
SORAMIC/Ricke	01126645	2019	III	BCLC C	SIRT + sorafenib Sorafenib	424	98 (23)	181 (42)	OS	Median OS combination arm: 12.1 *mo* Median OS control arm: 11.4 *mo* HR: 1.01; 95% CI: 0.81–1.25; **P: 0.9529**
He	02774187	2019	III	BCLC C with portal vein invasion	HAIC (oxaliplatin + fluorouracil+leucovorin) + sorafenib Sorafenib	247	80 (32)	247 (100)	OS	Median OS combination arm: 13.37 *mo* Median OS control arm: 7.13 *mo* HR: 0.35; 95% CI: 0.26–0.48; ***p* < 0.001** Median PFS combination arm: 7.03 *mo* Median PFS control arm: 2.6 *mo* HR: 0.33; 95% CI: 0.25–0.43; ***p* < 0.001** Any AEs combination arm: 118 [95.16%] Any AEs control arm: 109 [90.08%] **P: 0.15**
SIRveNIB/Chow	01135056	2018	III	BCLC B or C without extrahepatic disease	SIRT Sorafenib	360	0 (0)	110 (30)	OS	Median OS SIRT arm: 8.8 *mo* Median OS sorafenib arm: 10.0 *mo* HR: 1.1; 95% CI: 0.9–1.4; **P: 0.36**
Trial/First Author	NCT	Year	Phase	Clinical scenario	Treatment arms	N	EHS (%)	VI (%)	Primary end point	Outcome
STAH/Park	01829035	2018	III	Stages III, IVa, or IVb HCC according to the mUICC	cTACE + sorafenib Sorafenib	339	121 (36)	96 (28)	OS	Median OS combination arm: 12.8 mo Median OS control arm: 10.8 mo HR: 0.91; 90% CI: 0.69–1.21; P: 0.290 Median TTP combination arm: 5.3 mo Median TTP control arm: 3.5 mo HR: 0.67; 90% CI: 0.53–0.85; P: 0.003 Median PFS combination arm: 5.2 mo Median PFS control arm: 3.6 mo HR: 0.73; 90% CI: 0.59–0.91; P: 0.01 Severe AEs cTACE + Sorafenib: 33.3% Severe AEs Sorafenib: 19.8 % P: 0.006
Peng	Na	2018	Retrospective	BCLC C with initial hepatectomy	TACE + RFA+sorafenib Sorafenib	207	38 (18)	176 (85)	OS TTP	Median OS combination arm: 14.0 mo Median OS control arm: 9.0 mo *p* < 0.001 TTP combination arm: 7.0 mo TTP control arm: 4.0 mo *p* < 0.001
SILIUS/Kudo	01214343	2018	III	BCLC C Not suitable for resection, local ablation, or transarterial chemoembolization	HAIC (cisplatin + fluorouracil) + sorafenib Sorafenib	206	53 (25)	148 (59)	OS	Median OS combination arm: 11.8 mo Median OS control: 11.5 mo HR: 1.009; 95% CI: 0.743–1.371; P: 0.955 Median TTP combination arm: 5.3 mo Median TTP control arm: 3.5 mo HR: 0.645; 95% CI: 0.477–0.872; P: 0.004 Median PFS combination arm: 4.8 mo Median PFS control arm: 3.5 mo HR: 0.753; 95% CI: 0.566–1.003; P: 0·051
SARAH/Vilgrain	01482442	2017	III	BCLC C without extrahepatic disease	SIRT Sorafenib	459	277 (60)	0 (0)	OS	Median OS SIRT arm: 8.0 mo Median OS sorafenib arm: 9.9 mo HR: 1.15;95% CI: 0.94–1.41; P: 0.18
Duffy	01853618	2017	II	BCLC C with disease amenable to subtotal ablation in addition to having progressed on or been intolerant of prior sorafenib or BCLC B treated with TACE	Tremelimumab + subtotal RFA or CA Tremelimumab + subtotal TACE	32	12 (38)	Na	Safety Feasibly	No dose-limiting toxicities encountered Accumulation of intratumoral CD8 + T cells Median TTP TACE arm: 7.4 mo (95% CI: 2.2–19.4) Median TTP ablation arm: 7.4 mo (95% CI: 2.8- undefined) Median TTP Total: 7.4 mo (95% CI: 4.7–9.4) Median OS TACE arm: 13.6 mo (95% CI: 7.5 – undefined) Median OS ablation arm: 10.1 mo (95% CI: 4.6–15.7) Median OS Total: 12.3 mo (95% CI: 9.3–15.4)
Ikeda	Na	2016	II	BCLC C Not suitable for resection, local ablation, or transarterial chemoembolization	HAIC (cisplatin) + sorafenib Sorafenib	108	32 (30)	57 (53)	OS	Median OS combination arm: 10.6 mo Median OS control arm: 8.7 mo HR: 0.60; 95% CI: 0.38–0.96; P: 0.031
De Stefano	Na	2015	I	BCLC B or BCLC C	RFA + sorafenib Sorafenib	44	Na	Na	TTP Duration of sorafenib therapy	TTP combination arm: 10.3 mo (1-32) TTP control arm: 7.2 mo (0–38) Duration of sorafenib therapy combination arm: 10.9 mo (1-32) Duration of sorafenib therapy sorafenib arm: 7.3 mo (0–38)
Hu	Na	2014	Retrospective	BCLC C	TACE + sorafenib TACE	280	148 (53)	193 (69)	OS TTP	Median OS combination arm: seven mo Median OS control arm: 4.9 mo HR: 0.65; 95% CI: 0.49–0.87; P: 0.0003 TTP combination arm: 2.6 mo TTP control arm: 1.9 mo HR: 0.62; 95% CI: 0.47–0.82; P: 0.001

AEs: Adverse effects chemoembolization; BCLC: Barcelona-Clinic-Liver Cancer; DoR: Duration of reponse; HCC: Hepatocellular carcinoma; MWA: Micro-wave ablation; N: Number of patients; Na: Not applicable; NCT: National Clinical Trial; ORR: Objective response rate; OS: Overall survival; PFS: Progression free survival; TACE: Transarterial chemoembolization; TAE: Transarterial embolization; TARE: Transarterial radioembolization; TTP: Time-to-progression.

**Table 6.** Ongoing trials on LRTs and SYST combinations for stage (C)

Trial/First author	NCT	Phase	Clinical scenario	Treatment arms	N	Primary end point	Outcome
STOP-HCC	01556490	III	BCLC C	SIRT + sorafenib Sorafenib	526	OS	In progress
Na	03259867	II	BCLC C	TATE + nivolumab *or* pembrolizumab	80	ORR	In progress
NA	03099564	II	BCLC C	SIRT + pembrolizumab	30	PFS	In progress
Na	04483284	II	BCLC B or C ineligible for curative therapy	TACE + camrelizumab	60	PFS	In progress
Na	04479527	II	Patients with BCLC B and C with ⩾ 7 HCC *or* HCC > 5.0 cm Ineligible for surgery *or* local ablation	cTACE *or* DEB-TACE + HAIC-FOLFOX + camrelizuab + apatinib	34	PFS	In progress
Na	03592706	II/III	BCLC B or C	TACE + CIK	60	PFS	In progress
Na	03864211	I/II	BCLC C HCC ⩽ 10.0 cm	Toripalimab + RFA *or* MWA Toripalimab	147	PFS	In progress
Na	04220944	I	BCLC B or C HCC ⩾ 5.0 cm	MWA + TACE+sintilimab	45	PFS	In progress

BCLC: Barcelona-Clinic-Liver Cancer; SIRT: Selective Internal Radiotherapy Therapy ;OS: Overall survival; ORR: Objective response rate; TATE: Transarterial Tirapazamine Embolization; PFS: Progression free survival; TACE: Transarterial chemoembolization ; cTACE: Conventional transarterial chemoembolization ; DEB-TACE: Drug-eluting bead-transarterial chemoembolization; HAIC: Hepatic arterial infusion chemotherapy; CIK: Cytokine-induced killer; RFA: Radiofrequency ablation ; MWA: Micro-wave ablation ; n: Number of patients; Na: Not applicable applicable ; HCC: Hepatocellular carcinoma; TARE: Transarterial radioembolization;

#### Terminal stage (D)

The impact of LRTs combined with systemic therapies is yet to be explored in this population.

## Intrahepatic cholangiocarcinoma

CCA represents a heterogeneous group of malignancies arising from the biliary tract, the second most common primary liver cancer and are commonly classified as either perihilar, distal, or intrahepatic (iCCA).^3,79^ The carcinogenesis of CCA is yet to be fully understood. It often arises in biliary tissue characterized by chronic inflammation and cholestasis, with a resulting mixture of proinflammatory cytokines, growth factors, and bile acids. The presence of such complex components in the TME ultimately leads to increased and/or aberrant activation of various cell surface receptors, and to the down-regulation of intracellular signaling, all of which contribute to the carcinogenesis process.^80^ Similar to HCC, 70% of CCA cases are diagnosed at a metastatic or locally advanced stage, mainly due to the lack of specific symptoms during early stages.^81^ Current guidelines suggest Cisplatin-Gemcitabine (CisGem) as a first-line treatment for unresectable CCA cases, with a median OS of 11.4 months.^82^ The identification of isocitrate dehydrogenase-1 (IDH-1) mutations and fibroblast growth factor receptor-2 (FGFR-2) fusions as the most common targetable alternations in CCA have made them the subject of recent therapeutic interest.^83^ Indeed, selective FGFR inhibitors have been shown to be effective in patients with advanced refractory iCCA harboring the above-mentioned FGFR-2 alteration.^84–87^ Unsurprisingly, both Pemigatinib and Infigratinib have been approved by the FDA as second-line treatment options for refractory iCCA.^85^ Furthermore, the emergence of immunotherapy has prompted the initiation of several trials assessing the safety and efficacy of adaptive immune cell transfer or ICI in the context of iCCA treatment. Initial vaccine-based immunotherapies did not show improvements in clinical outcomes in iCCA patients.^88^ Nowadays, Pembrolizumab is indicated in iCCA patients with mismatch repair defects and microsatellite instability, as well as iCCA cases with a high mutational burden.^89^

LRTs can also be used as neoadjuvant treatment options in iCCA patients, mainly for tumor burden reduction and downstaging purposes in patients likely to undergo curative resection. This was demonstrated in the MISPHEC trial, which combined SIRT with CisGem as a first-line treatment in patients with unresectable iCCA, during which a significant proportion of patients were down-staged, allowing for surgical intervention.^90^ In addition, a recent Phase II trial by Cercek et al combining Gemcitabine plus Oxaliplatin and HAIC with floxuridine in unresectable iCCA patients has demonstrated an achieved OS of 25 months, rendering the combination as a suitable first-line treatment option.^91^

It is evident that the management of iCCA will keep evolving, thanks to the multiple ongoing trials (KEYNOTE-966, KEYNOTE-158…) involving SYST and/or LRTs Tables 7; 8 and particularly, the combinations of each. A recent Phase III trial, TOPAZ-1,^92^ showed a significantly longer median OS for with the combination of durvalumab, gemcitabine and cisplatine *vs* placebo (12.8 *vs* 11.1 months, *p* = 0.021). This result could pave the way for a new combination with LRTs.

**Table 7.** Trials on LRTs and SYST combinations for iCCA

Trial/First author	NCT	Year	Phase	Clinical scenario	Treatment arms	N	Primary end point	Outcome
MISPHEC/Edeline	Na	2020	II	Unresectable ICCA	SIRT + cisplastin+gemcitabine	41	RR	RR at 3 months: 39% (90% CI, 26–53) DCR at 3 months: 98% Median PFS: 14 *mo* (95% CI, 8–17) Median OS: 22 *mo* (95% CI, 14–52) 9 patients (22%): downstaged to surgical intervention
Cercek	01862315	2020	II	Unresectable ICCA	HAI (floxuridine) + gemcitabine+oxaplitatin	38	PFS	Median PFS: 11.8 *mo* (1-sided 90% CI, 11.1) At 6 months PFS: 84.1% (90% CI, 74.8%-infinity) Median OS: 25.0 *mo* (95% CI, 20.6-not reached) 1 year OS: 89.5% (95% CI, 80.2–99.8%)
Gangi	Na	2018	Retrospective	Unresectable ICCA	Chemotherapy *or* irradiation *or* surgical resection + SIRT SIRT	85	OS	Median OS from diagnosis: 21.4 *mo* (95% CI: 16.6–28.4) 3-, 6-, 12-, and 18 months OS from diagnosis: 98%, 94%, 83%, and 56% Median OS from first SIRT: 12.0 *mo* (95% CI: 8.0–15.2) 3-, 6-, 12-, and 18 months OS from first SIRT : 88%, 70%, 49%, and 38%

DEB-TACE: Drug-eluting bead-transarterial chemoembolization; TACE: Transarterial chemoembolization; CIK: Cytokine-induced killer; RFA: Radiofrequency ablation; Na: Not applicable; ORR: Overall response rate; SIRT: Selective Internal Radiotherapy Therapy ICCA: intrahepatic cholangiocarcinoma, N: number of patients; OS: Overall survival; PFS: Progression free survival; HAI: Hepatic arterial infusion; CA: Cryoablation.

**Table 8.** Ongoing trials on LRTs and SYST combinations for iCCA

Trial/First author	NCT	Phase	Clinical scenario	Treatment arms	N	Primary end point	Outcome
SIRCCA	02807181	III	Unresectable ICCA	SIRT + cisplatin+gemcitabine cisplatin + gemcitabine	89	OS	In progress
IMMUWHY	04238637	II	Unresectable ICCA	SIRT + durvalumab+tremelimumab SIRT + durvalumab	50	ORR	In progress
chol001	02482454	III	Unresectable ICCA	RFA + CIK RFA	50	RFS	In progress
IRB00233351	4301778	II	Unresectable ICCA	TACE *or* SIRT + carvalumab+CSF-1R inhibitor	30	ORR	In progress
Na	05124002	IV	Unresectable ICCA	HAI (FOLFOX) + Recombinant Human Adenovirus Type five intratumorally	66	PFS	In progress
GHX	04834674	II	Unresectable ICCA Stand first-line chemotherapy resistance	DEB-TACE + apatinib+PD-1 antibody	20	ORR PFS	In progress
Na	04299581	II	Unresectable ICCA Failed of 1 line of systemic regimens	CA + camrelizumab	25	ORR	In progress

DEB-TACE: Drug-eluting bead-transarterial chemoembolization; TACE: Transarterial chemoembolization; CIK: Cytokine-induced killer; RFA: Radiofrequency ablation; Na: Not applicable; ORR: Overall response rate; SIRT: Selective Internal Radiotherapy Therapy ICCA: intrahepatic cholangiocarcinoma, N: number of patients; OS: Overall survival; PFS: Progression free survival; HAI: Hepatic arterial infusion; CA: Cryoablation.

Despite all this, relapse rates in iCCA remain far high and prognosis far too low. Hence, the need for further exploration in neoadjuvant and adjuvant combinatorial strategies, as well as a better method of patient selection, in the hope of improving long-term oncological outcomes.

### Secondary liver cancer

Metastatic disease is the most frequent form of liver malignancies and cancer recurrence,^93^ and is often associated with a poor prognosis despite all therapeutic advances.^94^ Interestingly, even for cancers responding to immunotherapies, liver involvement is considered as an independent factor of poor prognosis and poor response. In this setting, LRTs, in combination with other systemic approaches, have shown promises in terms of improving clinical outcomes.^95–97^ Unlike chemotherapy, which has witnessed a decline due to a high rate of morbidity and local recurrence, LRTs are incrementally gaining practice as first-line, second-line, or adjuvant treatment options for liver metastases to improve the OS or even allow a down-staging for surgery.^98^

### Colorectal carcinoma liver metastases

Colorectal cancer (CRC) is the third most common malignancy, the leading cause of liver metastasis (LM) and the second most deadly cancer.^99^ Approximately, half of CRC patients experience synchronous or metachronous metastatic liver disease, with only one quarter of patients being eligible for surgical intervention.^100^ The standard of care is currently a combination of chemotherapy, most commonly FOLFOX or FOLFIRI, in combination with targeted therapies, mainly Bevacizumab or Cetuximab, with mFOLFOX and Bevacizumab combination being approved for clinical use.^101^

In a study by Pilati et al, in order to combine first-line therapeutics with LRTs, patients with unresectable LM received either Floxuridine-Leucovorin HAI alone or in combination with systemic chemotherapy. There were no differences between the two arms, with ORRs and OS at 52.7% and 18.0 months in HAI alone arm compared to 50.6% and 19.1 months in the combination group (*p* > 0.05).^102^

Recently, the OPTILIV trial demonstrated a 15% increase of conversion rate to surgery in patients with previously unresectable LM when combining systemic Cetuximab with Irinotecan-Oxaliplatin-5-FU HAI.^103^ Two other trials using modern systemic chemotherapy in combination with HAI demonstrated similar results.^104,105^ Further RCT are required to confirm such improvements. Luckily, the Phase III PUMP (NTR7493), and PACHA-01 (NCT02494973) trials are assessing the role of HAI as an adjuvant therapy to surgical resection in CRC-LM patients.

As for ablation therapies, the recent Phase II CLOCC trial demonstrated an improvement of OS when combining RFA with systemic chemotherapy, with a median OS of 45.6 months in patients receiving combined treatment *vs* 40.5 months in patients only treated with chemotherapy (*p* = 0.01).^106^ As of today, there are no current trials evaluating the combination of MWA and systemic therapy in CRC-LM patients.

Regarding immunotherapy, it has been well established that ICI can be highly effective in mismatch repair deficient (dMMR) and microsatellite instability-high (MSI) CRC cancers. This is in part due to the associated high tumor mutational burden and consequential increased immunogenicity, abundant expression of IC regulators, and Immune cell infiltration.^107–109^ Indeed, Pembrolizumab has been recently shown to extend PFS by 8.3 months in dMMR/MSI-CRC patients, compared to patients receiving chemotherapy alone.^110^ Unfortunately, only 2–4% of CRC cases are diagnosed as dMMR or MSI.^111^ However, similar to the rationale in HCC, the immunological impacts of LRTs in CRC-LM are anticipated, and synergy between IO approaches and immunotherapy is being considered. Following this logic, *Lemdami et al* had demonstrated an increase in specific T cell response, and significant tumor regression in CRC-LM patients receiving RFA in combination with *in-situ* immunotherapy or PD1/PDL1 blockade as adjuvant therapy.^112^ In addition, an increase in tumor antigen specific T cell infiltration and PDL1 expression in primary CRC tumors was observed in CRC-LM patients receiving RFA.^113^ Further explorations of the potential synergy of RFA and PD1/PDL1 blockade are required.

As for alternatives to current systemic therapies, DEB-TACE combined with Irinotecan (DEBIRI) resulted in a median OS and PFS for 22 and 7 months, a significant improvement from a median of OS and PFS of 15 and 4 months in FOLFIRI treated CRC-LM patients in addition to quality-of-life amelioration. Moreover, further analysis demonstrated a greater benefit for KRAS-wild type patients within the DEBIRI treatment arm, with an OS of 26 months, compared to 14 months in KRAS-mutated patients.^114^ Furthermore, significant improvements in ORRs, hepatic PFS, and a more durable overall PFS were observed when combining DEBIRI with FOLFOX, compared to FOLFOX alone, in patients undergoing down-staging chemotherapy.^115^ Similarly, the PARAGON-II trial asserted that DEBIRI was comparable to systemic neoadjuvant chemotherapy in terms of OS and anti tumor response.^116^ Despite these encouraging results, more data is required from RCT prior to enlisting such combinations per guideline Tables 9; 10.

**Table 9.** Trials on LRTs and SYST combinations for CRC

Trial/First author	NCT	Year	Phase	Clinical scenario	Treatment arms	N	Primary end point	Outcome
EPOCH/Mulcahy	01483027	2021	III	Unresectable LMCRC Progression after first-line chemotherapy based on irinotecan or oxaliplatin	SIRT + chemotherapy+/- targeted therapy Chemotherapy ± targeted therapy	428	PFS hepatic PFS	Median PFS combination arm: 8.0 *mo* Median PFS control arm: 7.2 *mo* HR: 0.69; 95% CI: 0.54–0.88); **P: 0.0013** Median hPFS combination arm: 9.1 *mo* Median hPFS chemotherapy alone arm: 7.2 *mo* HR: 0.59; 95% CI: 0.46–0.77; ***p* < 0.0001** Median OS combination arm: 14.0 *mo* Median OS control arm: 14.4 *mo* HR: 1.07; 95% CI: 0.86–1.32); **P: 0.7229**
FOXFIRE/Winter	83867919	2019	III	Unresectable or untreatable local abaltion of LMCRC	OxMdG ± surgery SIRT + OxMdG +/- surgery	364	OS	Resection rate SIRTcombination arm: 21% Resection rate control arm: 18%, **P: 0.508** Median time-to-resection SIRT combination arm: 8.3 mo (95% CI: 7.4–9.9) Median time-to-resection control arm: 7.8 *mo* (95% CI: 6.5–8.5) h: 0.94; 95% CI: 0.58–1.53; **P: 0.810** Median time from resection SIRT combination arm: 21.9 *mo* (95% CI: 19.0–35.7) Median time from resection to death control arm: 25.2 *mo* (95% CI: 21.0-not estimated) HR: 1.55; 95% CI: 0.83–2.89; **P: 0.164**
REsect-SIRFLOX/Garlipp	00724503	2019	Post hoc	Unresectable LMCRC chemotherapy-naive	SIRT + FOLFOX+/- bevacizumab FOLFOX ± bevacizumab	472	Change in technical resectability of all liver lesions between baseline and nadir	Difference in resectability combination arm: 38.1% (93 patients) Difference in resectability control arm: 28.9% (66 patients) RR: 1.25; 95% CI: 1.10–1.54; ***p* < 0.001**
Pak	00492999	2018	II	Unresectable LM-CRC without extrahepatic disease	HAI (Floxuridine) + chemotherapy+/- bevacizumab	64	Complete resection	32 patients (52%): conversion to resection at median of 5 *mo* (2-22) Median PFS: 13 *mo* Median OS from initial diagnosis: 46 *mo* (32.3–59.7) Median OS from treatment initiation: 38 *mo* (28.8–53.7) 5 year OS: 35.8% (24.3–47.5)
Di Noia	NA	2017	II	Liver-limited or liver-dominant disease, who have failed at least two previous lines of chemotherapy	DEBIRI + capecitabine	40	ORR	Median PFS: four *mo* Median OS: eight *mo*
CLOCC/Ruers	00043004	2017	II	Unresectable LMCRC without extrahepatic disease	RFA + Systemic treatment ± resection Systemic treatment ± resection	119	OS	3-, 5- and 8 year OS combination arm: 56.9%, 43.1 and 35.9% 3-, 5-, and 8 year OS control arm: 55.2%, 30.3 and 8.9% Median OS combination arm: 45.6 *mo* Median OS control arm: 40.5 *mo* HR: 0.58; 95% CI: 0.38–0.88; **P: 0.01** Median PFS combination arm: 16.8 *mo* Median PFS control arm: 9.9 *mo* 3-, 5- and 8 year PFS combination arm: 27.7%, 24.2 and 22.3% 3-, 5- and 8 year PFS control arm: 11.9%, 5.9 and 2.0% h: 0.57; 95% CI: 0.38–0.85; **P: 0.005**
Trial/First author	NCT	Year	Phase	Clinical scenario	Treatment arms	N	Primary end point	Outcome
SIRFLOX/Van Hazel	00724503	2016	III	Unresectable LMCRC and chemotherapy-naive	SIRT + FOLFOX+/- bevacizumab FOLFOX ± bevacizumab	530	PFS	Median PFS combination arm at any site: 10.7 mo Median PFS control arm at any site: 10.2 mo HR: 0.93; 95% CI: 0.77–1.12; P: 0.43 PFS in the liver combination arm: 20.5 mo PFS in the liver control arm: 12.6 mo HR: 0.69; 95% CI: 0.55–0.90; P: 0.002 ORR combination arm: 76.4% ORR control arm: 68.1% P: 0.113
Li	02419677	2016	II/III	Unresectable LMCRC without extrahepatic disease and ⩽5 LMCRC and LMCRC ⩽ 5.0 cm	RFA + CIK RFA	60	PFS	Median PFS combination arm: 23 mo Median PFS control arm: 18.5 mo P: 0.0336 Median OS combination arm: not reached Median OS control arm: 43 mo 3 year survival rates RFA: 64.6% 3 year survival rates RFA + CIK: 81.0% P: 0.1187
D'Angelica	00492999	2015	II	Unresectable LMCRC without extrahepatic disease	HAI (Floxuridine) + chemotherapy+/- bevacizumab	49	Conversion to resection	Conversion to resection at a median of 6 mo (3-22) from treatment initiation: 23 patients (47%) Median OS: 38 mo (28-not reached) Median PFS: 13 mo (7-16)
Martin	G080230-S001	2015	II	Unresectable LMCRC	DEBIRI + mFOLFOX+/- bevacizumab mFOLFOX ± bevacizumab	72	ORR	2-, 4- and 6 months ORR combination arm: 78%, 95 and 76% 2-, 4- and 6 months ORR control: 54%, 70 and 60% Median PFS combination arm: 12 mo (9–15.4) Median PFS control: 15.3 mo (10.4–20) P: 0.18 Median hepatic PFS combination arm: 17 mo (12-23) Median hepatic PFS control arm: 12 mo (11-24) P: 0.05
OPTILIV/Levi	00852228	2015	II	Unresectable LMCRC	HAI (iritotecan, oxaplatin and 5-fluoroucacil) + cetuximab + surgery	64	LM rate of macroscopic complete resections (R0 + R1)	Median PFS total: 9.3 mo (7.8–10.9) Median PFS patients undergo complete resection: 15.7 mo (12.7–18.7) Median PFS others patients: 8.6 mo (6.4–10.9) P < 0.001 Median OS total: 25.5 mo (18.8–32.1) Median OS patients undergo complete resection: 35.2 mo (32.6–37.8) Median OS others patients: 18.7 mo (12.1–25.3) P < 0.001
Hendlisz	Na	2010	III	LM-CRC unresectable to curative surgery or local ablation and chemotherapy-refractory (FU, oxaliplatin, and irinotecan)	SIRT + fluorouracil Fluorouracil	44	Time to liver progression	Median TTLP combination arm: 5.5 mo Median TTLP control arm: 2.1 mo HR: 0.38; 95% CI: 0.20–0.72; P: 0.003 Median TTP combination arm: 4.5 mo Median TTP control arm: 2.1 mo HR: 0.51; 95% CI: 0.28–0.94; P: 0.03
Van Hazel	Na	2009	I	LMCRC not treatable and that were refractory to prior fluorouracil chemotherapy	SIRT + irinotecan	25	Maximum-tolerated dose	Median PFS: 6.0 mo (1.6–11.4) Median hepatic PFS: 9.2 mo (1.6–25.8) Median survival: 12.2 mo (2.8–60)
Pilati	Na	2009	Retrospective	Unresectable LMCRC without extrahepatic disease	HAI (floxuridine + leucovorin) + 5-fluorouracil + leucovorin or HAI	153	OS	Median OS combination arm: 18 mo Median OS control arm: 19.1 mo *p* > 0.05

TTP: Time to progression; LMCRC: Liver metastases Colorectal carcinoma; DEBIRI: Drug eluting beads loaded with irinotecan; Na: Not applicable; SIRT: Selective Internal Radiotherapy Therapy; N: number of patients; OS: Overall survival; PFS: Progression free survival; HAI: Hepatic arterial infusion; RFS: Recurrence free survival; ORR: Overall response rate; TTLP: Time to liver progression; OxMdG: oxaliplatin, 5-fluorouracil and folic acid; LTP: Local tumor progression.

**Table 10.** Ongoing trials on LRTs and SYST combinations for CRC

Trial/First author	NCT	Phase	Clinical scenario	Treatment arms	N	Primary end point	Outcome
EMBOBEVA/Fiorentini	03732235	Case-contol	Unresectable LMCRC	DEBIRI + bevacizumab FOLFIRI + bevacizumab	50	TTP	In progress
SIRTCI	04659382	II	Liver-dominant disease with up to six extrahepatic lesions Unresectable LMCRC	Capecitabine + oxaplatin+bevacizumab + atezolizumab+ SIRT	52	PFS	In progress
iRE-C	04108481	I/II	pMMR/MSS Unresectable LMCRC	SIRT + durvalumab	18	Maximum tolerated dose	In progress
Na	04888806	II	Unresectable LMCRC	Camrelizumab + MWA+chemotherapy	37	PFS	In progress
PACHA-01	02494973	III	Curative-intent of resection *or* ablation R0 of ⩾ 4 LMCRC	Oxaliplatin IV + mFOLFOX6 HAI (oxaliplatin) + LV5FU2	220	18 month hepatic RFS rate 3 year RFS rate	In progress

TTP: Time to progression; LMCRC: Liver metastases Colorectal carcinoma; DEBIRI: Drug eluting beads loaded with irinotecan; Na: Not applicable; SIRT: Selective Internal Radiotherapy Therapy; N: number of patients; OS: Overall survival; PFS: Progression free survival; HAI: Hepatic arterial infusion; MSS: Microsatellite stable; pMMR: Proficient mismatch repair; MWA: Microwave ablation; RFS: Recurrence free

TARE has also been proven efficacious in CRC patients with LM ineligible for resection, ablation, or further systemic therapies. For instance, the retrospective MORE study had demonstrated a survival benefit, with an OS of 10 months.^117^ Combining TARE with fluorouracil as a first-line treatment option resulted in an overall PFS of 4.5 months and a liver PFS of 5.5 months, compared to 2.1 months as PFS and liver PFS in fluorouracil only treated patients, without differences in OS.^118^ SIRLFOX trial noted a comparable PFS benefit when combining TARE with FOLFOX, with or without Bevacizumab, as well as drastically delaying local progression.^119^ Furthermore, the following post hoc analysis revealed a statistically significant increase in the eligibility for surgical resection in patients receiving SIRT, compared to the control group,^119^ suggesting a more effective downstaging strategy when combining SIRT with systemic chemotherapy in CRC-LM patients. Besides, Mulcahy et al has demonstrated that the addition of TARE to second-line chemotherapy had extended both PFS and hepatic PFS (8 and 9.1 months, *p* = 0.0013 and *p* < 0.0001 respectively) compared to the chemotherapy alone treatment group.^120^ In this context, there is further need for better patient selection prior to incorporating TARE into second-line treatment options in CRC patients with unresectable LM. With the help of LRTs and advancements in down-staging approaches, patients with LM disease are becoming more readily eligible for surgical intervention, and are consequently experiencing improvements in oncological end points. The inclusion of DEBIRI and SIRT into guidelines, either alone or in combination with well-established SYST, has resulted in better management and survival for LM patients.

## Others

Local and systematic treatments are under evaluation for liver metastases of other primary cancers such as neuroendocrine (NCT02859064, NCT03457948), breast tumors^121^ or uveal melanoma (NCT04463368).

### Conclusion

In conclusion, locoregional image-guided therapies can stimulate the immune response and/or allow a pause in systemic treatments. Recent studies show promising results regarding their combination with systemic treatments. Although these combination treatments are still being evaluated, with mostly early phase trials published to date, this innovative approach is expected to play an increasing role in patient management, particularly in the era of “personalized care”. In this context, multidisciplinarity is key to define the best therapeutic strategy to help achieve a durable oncological response, including the types of combinations, the right therapeutic sequence, and the appropriate choice of targets.

Even if it is too early to give a clear answer to the title of this review, recent trials suggest that image-guided locoregional therapies and new systemic treatments tend to be new allies. In order to develop a sustainable friendship, carefully designed large-scale randomized trials are needed, with a particular attention regarding patient selection.
